# Structure-Function Features of a Mycoplasma Glycolipid Synthase Derived from Structural Data Integration, Molecular Simulations, and Mutational Analysis

**DOI:** 10.1371/journal.pone.0081990

**Published:** 2013-12-03

**Authors:** Javier Romero-García, Carles Francisco, Xevi Biarnés, Antoni Planas

**Affiliations:** Laboratory of Biochemistry, Institut Químic de Sarrià, Universitat Ramon Llull, Barcelona, Spain; University of Michigan, United States of America

## Abstract

Glycoglycerolipids are structural components of mycoplasma membranes with a fundamental role in membrane properties and stability. Their biosynthesis is mediated by glycosyltransferases (GT) that catalyze the transfer of glycosyl units from a sugar nucleotide donor to diacylglycerol. The essential function of glycolipid synthases in mycoplasma viability, and the absence of glycoglycerolipids in animal host cells make these GT enzymes a target for drug discovery by designing specific inhibitors. However, rational drug design has been hampered by the lack of structural information for any mycoplasma GT. Most of the annotated GTs in pathogenic mycoplasmas belong to family GT2. We had previously shown that MG517 in *Mycoplasma genitalium* is a GT-A family GT2 membrane-associated glycolipid synthase. We present here a series of structural models of MG517 obtained by homology modeling following a multiple-template approach. The models have been validated by mutational analysis and refined by long scale molecular dynamics simulations. Based on the models, key structure-function relationships have been identified: The N-terminal GT domain has a GT-A topology that includes a non-conserved variable region involved in acceptor substrate binding. Glu193 is proposed as the catalytic base in the GT mechanism, and Asp40, Tyr126, Tyr169, Ile170 and Tyr218 define the substrates binding site. Mutation Y169F increases the enzyme activity and significantly alters the processivity (or sequential transferase activity) of the enzyme. This is the first structural model of a GT-A glycoglycerolipid synthase and provides preliminary insights into structure and function relationships in this family of enzymes.

## Introduction

Mycoplasmas, obligate parasites associated with persistent infections, are characterized by their minute size and total lack of a cell wall, which is used to separate taxonomically mycoplasmas from other bacteria in the class *Mollicutes* [1–3]. Membranes of mycoplasma contain free glycoglycerolipids as structural elements with a fundamental role in membrane properties and stability. Monoglycosyldiacylglycerol and diglycosyldiacylglycerol are the major glycolipids in mycoplasma membranes, where their nonbilayer-bilayer balance contributes to membrane properties such as curvature and stability, as shown in *Acholeplasma laidlawii*, one of the best investigated bacteria with regard to the function of glycolipids in biological membranes [4–6]. 

Glycoglycerolipids are synthesized by glycosyltransferases (GTs) that catalyze the glycosyl transfer from a sugar nucleotide donor to diacylglycerol as acceptor [7]. The large diversity of glycosyltransferases (EC2.4.1.x) are classified based on sequence similarities into closely 100 different families in the CAZy database (www.cazy.org) [8,9], which contains about 87.000 entries with more than 90% being uncharacterized open-reading frames. Structural information from X-ray crystallography is only available for over 100 GTs in 38 GT families, and covers both eukaryotic and prokaryotic origins as well as inverting and retaining transferase mechanisms [10,11]. In contrast to the large diversity of reactions catalyzed by GTs, mainly two general folds are observed in their structure: GT-A and GT-B. Both folds are related to the nucleotide-binding domain of the Rossmann-like fold type. The GT-A fold topology consists of a central continuous β-sheet surrounded by α-helices on both sides. The GT-B fold, instead, consists of two β/α/β Rossmann domains facing each other through a flexible link. Despite the structural homogeneity among members belonging to the same fold, there is no consensus sequence profile for the whole clan of GT families that define each fold. 

**Figure 1:**
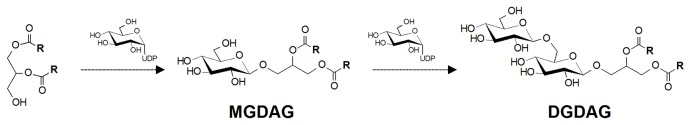
Reaction catalyzed by *Mycoplasma genitalium* GT MG517. It is a membrane-associated sequentially-acting GT activated by anionic phospholipids.

Among *Mollicutes*, the order with larger number of currently identified species is *Mycoplasmatales*, with the genus *Mycoplasma* having more than 100 species, many being human pathogens. Genome-sequenced mycoplasmas have a reduced number of annotated (putative) glycosyltransferases, consistent with their limited biosynthetic capabilities as a consequence of their reduced genomes evolved by degenerative or reductive evolution [1,2]. Most of the annotated glycosyltransferases in pathogenic mycoplasmas belong to family GT2, where just two orthologous GT2 processive enzymes from *M. pneumonia* [12] and *M. genitalium* [13] have been experimentally identified. The first is a causative agent of atypical pneumonia [14,15], whereas *M. genitalium* is involved in urogenital diseases such as acute and chronic non-gonococcal urethritis, cervicitis, and pelvic inflammation [16,17]. GTs synthesizing glycoglycerolipids have been proposed as potential therapeutic targets against mycoplasma infections [7]. We have previously shown that GT MG517 (glycosyldiacylglycerol synthase) is responsible for the biosynthesis of membrane glycoglycerolipids in *Mycoplasma genitalium*, with an essential function for mycoplasma viability [13]. It catalyzes the sequential transfer of glycosyl units from a sugar nucleotide donor (UDPGlc or UDPGal) to diacylglycerol (DAG) to form mono- and di-glycosyldiacylglycerols (Figure 1). The absence of glycoglycerolipids in animal host cells of mycoplasma infections makes this GT enzyme a target for drug discovery by designing specific inhibitors. However, rational drug design has been hampered by the lack of structure-function relationships for any mycoplasma GT. 

To date there is no solved crystal structure for any glycoglycerolipid (GGL) synthase. However, three dimensional models have been built for two types of GGL synthases of the GT-B fold: the glucosyldiacylglycerol synthases from *Acholeplasma laidlawii* and *Streptococcus pneumonia* [18] belonging to the GT4 family, and the monogalactosyldiacylglycerol synthase from *Spinacia aleracea* [19] that belongs to the GT28 family. All these homology-based models were essentially built taking *E. coli* MurG as structural template, which is the only GT28 structure currently available. On the other hand, no structural models have been reported for any GGL belonging to the GT-A fold.

Unsuccessful attempts to obtain a crystallographic structure of GT MG517 prompted us to build a three dimensional model structure of this GT-A family GT2 glycosyldiacylglycerol synthase by homology modeling and long scale molecular dynamics simulations. Multiple replicas of the model were generated by combining different templates guided by a novel sequence profile of the GT-A fold clan derived from sequence and structure comparisons. The GT MG517 model provides for the first time structural insights on a GT-A glycoglycerolipid synthase and the structural determinants of donor/acceptor specificity. Preliminary mutational analysis at selected residues provides evidences of their structure-function relationships and allows discriminating among model replicas.

## Results and Discussion

### Sequence and Structure analysis of GT-A fold glycosyltransferases

**Figure 2:**
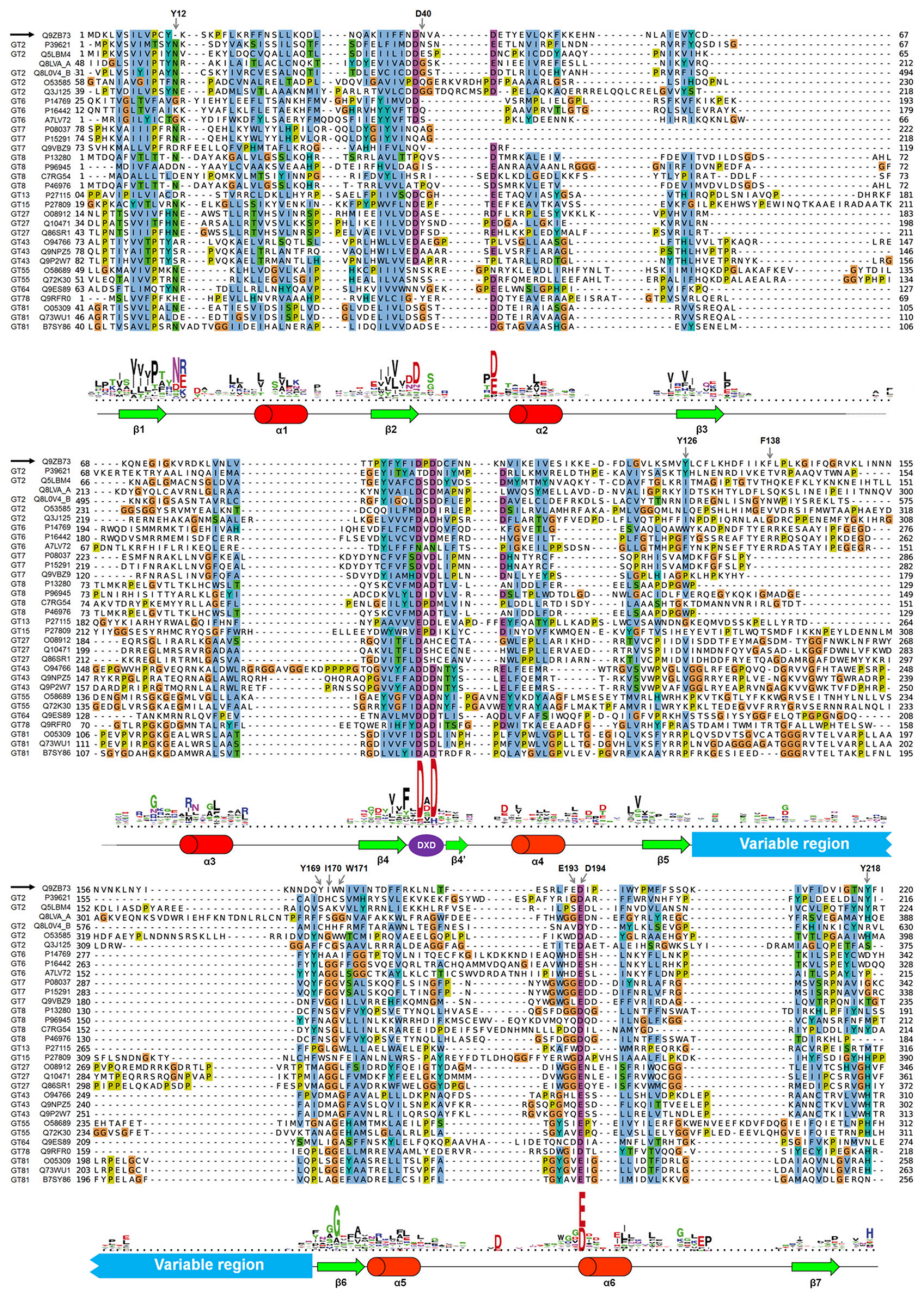
Sequence alignment of GT-A proteins with 3D structures solved by X-ray crystallography. Alignment logo, and consensus secondary structure are plotted below (full secondary structure alignment is detailed in Figure S1). MG517 sequence is aligned on the top (black arrow), and mutated residues are indicated by arrows.

The list of GT-A glycosyltransferases characterized so far, with available structural information, is reported in Table 1. This set of structures/sequences is formed by 30 glycosyltransferases of different origin and function which belong to 12 different GT families. All these proteins bear at least a glycosyltransferase catalytic domain, while some of them have two GT domains or additional domains of different function. The multiple sequence alignment of these GT-A sequences (GT domain Table 1, last column) together with the consensus secondary structure annotation is presented in Figure 2 (full secondary structure alignment in Figure S1). 

**Table 1:** GT-A enzymes with solved crystal structures in the PDB (June 2013)^*a*^.

**PDB**	**UniProt**	**Family**	**Organism / enzyme**	**Full length (aa)**	**GTA domain (aa)^*b*^**
1QG8	P39621	GT2	*Bacillus subtilis*. / Spore coat polysaccharide biosynthesis protein (SpsA)	256	1-216
3L7J **^*c*^**	Q5HLM5	GT2	*Staphylococcus epidermidis*. / Teichoic acid biosynthesis protein F	721	-
3BCV	Q5LBM4	GT2	*Bacteroides fragilis* (strain ATCC 25285 / NCTC 9343). / Putative glycosyltransferase protein	342	3-226
2Z86	Q8L0V4	GT2	*Escherichia coli*. / Chondroitin polymerase (two GT domains)	686	148-388 / 431-630
4FIY	O53585	GT2	*Mycobacterium tuberculosis* H37Rv. / Galactofuranosyl transferase GlfT2 (GalTr)	637	158-398
4HG6	Q3J125	GT2	*Rhodobacter sphaeroides*. / Possible cellulose synthase	788	139-375
1O7Q	P14769	GT6	*Bos taurus*. / N-acetyllactosaminide alpha‐1,3‐galactosyltransferase Galactosyltransferase (α3GalT)	368	125-342
3IOH	P16442	GT6	*Homo sapiens*. / Histo-blood group ABO system transferase	354	112-328
4AYL	A7LVT2	GT6	*Bacteroides ovatus*. / Glycosyltransferase family 6	263	1-215
1NKH	P08037	GT7	*Bos taurus*. / Beta-1,4‐galactosyltransferase 1 (b4Gal‐T1)	402	178-342
2FY7	P15291	GT7	*Homo sapiens*. / Beta-1,4‐galactosyltransferase 1 (b4Gal‐T1)	398	174-338
3LW6	Q9VBZ9	GT7	*Drosophila melanogaster*. / Beta-4-galactosyltransferase 7	322	73-235
1LL2	P13280	GT8	*Oryctolagus cuniculus*. / Glycogenin-1	333	1-191
1G9R	P96945	GT8	*Neisseria meningitidis*. / Glycosyl transferase	311	1-212
3TZT	C7RG54	GT8	*Anaerococcus prevotii.*/ Glycosyl transferase family 8	273	1-214
3T7O	P46976	GT8	*Homo sapiens* / Glycogenin-1	350	1-184
1FO8	P27115	GT13	*Oryctolagus cuniculus*. / Alpha-1,3‐mannosyl‐glycoprotein 2-beta-Nacetylglucosaminyltransferase (GlcNAc-T I)	447	104-316
1S4N	P27809	GT15	*Saccharomyces cerevisiae*. / Glycolipid 2-alpha-mannosyltransferase	442	119-390
1XHB	O08912	GT27	*Mus musculus*. / Polypeptide N-acetylgalactosaminyltransferase 1 (ppGaNTase-T1)	559	114-346
2FFU	Q10471	GT27	*Homo sapiens*. / Polypeptide N-acetylgalactosaminyltransferase 2 (ppGaNTase-T2)	571	134-361
2D7I	Q86SR1	GT27	*Homo sapiens*. / Polypeptide N-acetylgalactosaminyltransferase 10 (ppGaNTase-T10)	603	143-372
3CU0	O94766	GT43	*Homo sapiens*. / Galactosylgalactosylxylosylprotein 3-betaglucuronosyltransferase 3(GlcAT-I)	335	73-310
2D0J	Q9NPZ5	GT43	*Homo sapiens*. / Galactosylgalactosylxylosylprotein 3-betaglucuronosyltransferase 2(GlcAT-S)	323	78-302
1V84	Q9P2W7	GT43	*Homo sapiens*. / Galactosylgalactosylxylosylprotein 3-betaglucuronosyltransferase 1(GlcAT-P)	334	82-313
2ZU9	O58689	GT55	*Pyrococcus horikoshii*. / Mannosyl-3‐phosphoglycerate synthase (MPGS)	394	49-312
2WVL	Q72K30	GT55	*Thermus thermophilus*. / Mannosyl-3‐phosphoglycerate synthase (MpgS)	391	51-311
1OMZ	Q9ES89	GT64	*Mus musculus*. / Exostosin-like 2	330	63-274
2BO4	Q9RFR0	GT78	*Rhodothermus marinus* (*Rhodothermus obamensis*). / Mannosylglycerate synthase	397	1-218
3E26	O05309	GT81	*Mycobacterium tuberculosis*. / Glycosyl-3-phosphoglycerate synthase (GpgS)	324	41-258
3CKJ	Q73WU1	GT81	*Mycobacterium avium* subsp. *paratuberculosis* K-10. / Glycosyl-3-phosphoglycerate synthase (GpgS)	329	46-263
3O3P	B7SY86	GT81	*Rubrobacter xylanophilus* PRD-1. / Mannosyl-3‐phosphoglycerate synthase (MpgS)	387	40-256

*a* For those enzymes with more than one PDB file, the structure with highest resolution was taken.

*b* Amino acid residues corresponding to the GT domain and used in the sequence alignments

*c* The PDB file corresponds to the glycerophosphotransferase domain of Q5HLM5, whereas the glycosyltransferase domain is not solved.

**Figure 3:**
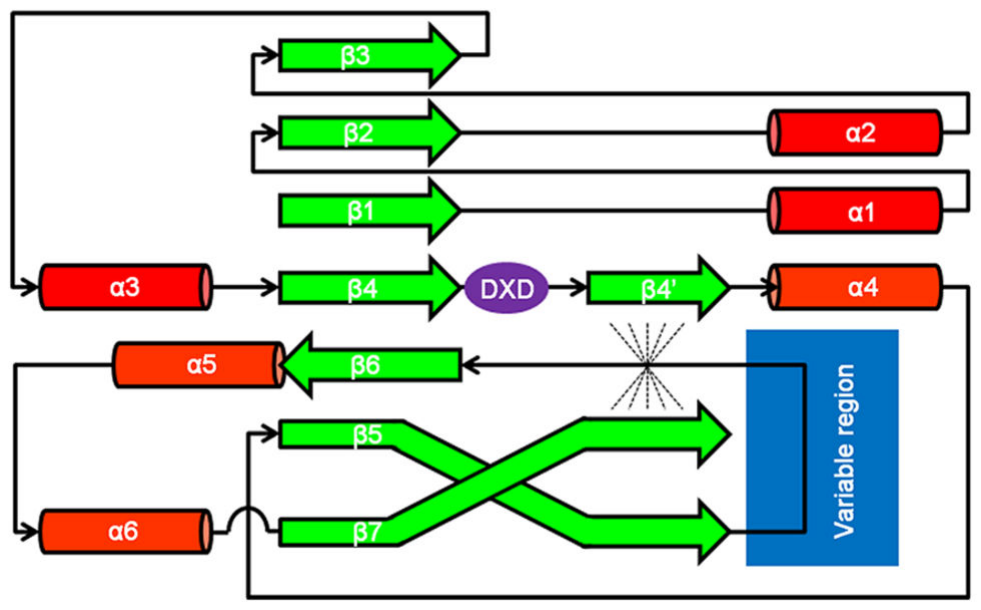
Consensus topology map for GT-A proteins. It is based on the structural superimposition of the 3D structures of solved GT-A enzymes in Table 1 (Figure S2).

When looking at the structural superimposition of GT-A structures (Figure S2), it is evident that the topology of all GT-A is highly conserved. The consensus topology of secondary structure elements (Figure 3) is formed by seven β-strands that form an extended and twisted β-sheet flanked by three α-helices at each side of the β-sheet platform. The conserved DXD motif is located in the center between β-strand 4 and α-helix 4. Interestingly, β-strands 5 and 7 cross each other in the structure; this allows the formation of a parallel platform of β-strands that extends up to the DXD motif (dashed structure in Figure 2). Not in all GT-A structures the β-strand 7 is fully resolved. In those cases, this parallel β-sheet platform is not formed, and the region between the DXD motif and helix 4 is unstructured. GT7 family deviates slightly from this conserved topology, where helix 2 and β-strand 3 are missing but replaced by a piece of structure located further in the sequence (data not shown). Nevertheless, the structural arrangement of secondary structure elements is highly conserved in all GT-A proteins. 

Particularly interesting is the region between conserved β-strand 5 and β-strand 6, where a highly variable region is detected (blue box in Figures 2 and 3). Neither a consensus structure nor a conserved amino acids pattern can be assigned to it, and this is probably the reason why a consensus sequence profile has not been previously reported for the GT-A fold clan. This region has previously been seen as the interfacing surface upon dimerization in this family of proteins [20] but no further implications have been discussed. The presence of this structurally variable region will difficult the generation of a model for the target protein MG517 as no consensus structure can be hypothesized *a priori*.

Because standard sequence alignment tools did not reproduce the structural superposition of GT-A sequences, the multiple sequence alignment reported in Figure 2 was manually adjusted by visual inspection of the superimposed structures (see methods). The curated multiple sequence alignment was then used to build a Hidden Markov Model (HMM) profile of the GT-A fold clan (available in the Information, File S1). Although the variable region could not be incorporated into the profile, the HMM captures both conserved regions of the alignment that flank the variable region. In this way, the profile allows detecting and aligning properly these regions in any member of the GT-A fold clan of proteins. 

**Figure 4:**
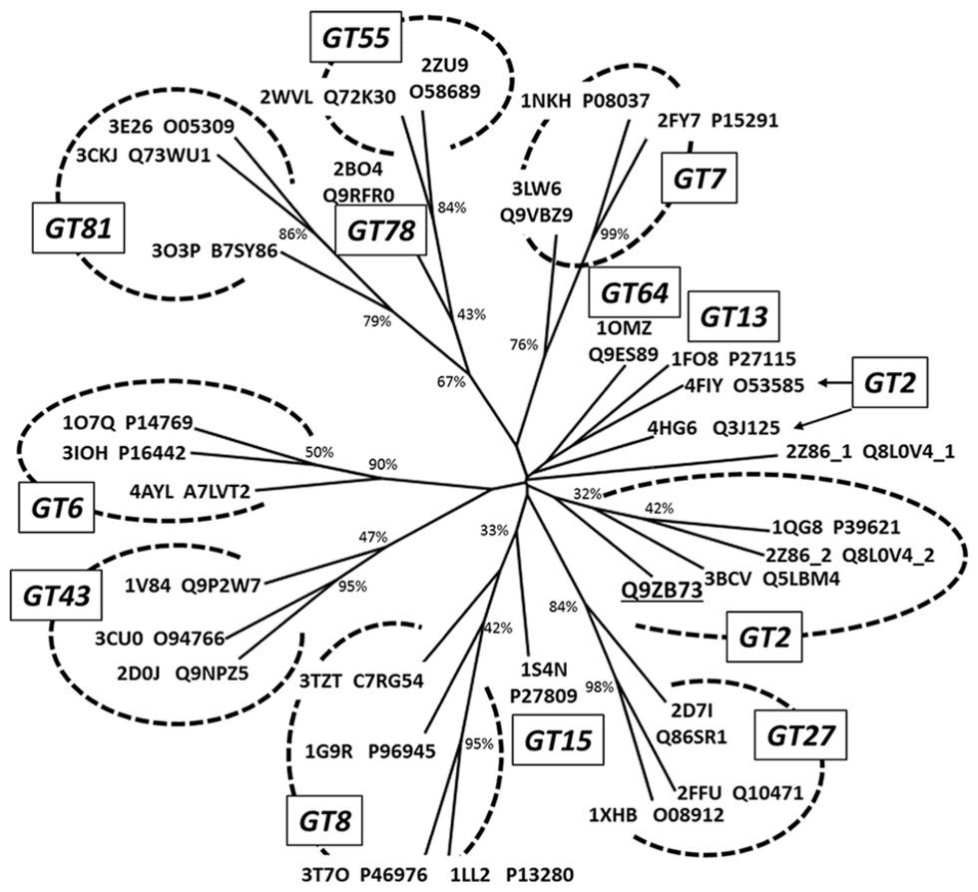
Phylogenetic tree of GT-A proteins with known 3D structure. The tree was generated from the curated GT domain multiple sequence alignment shown in Figure 2. Proteins are labeled with their PDB and UNIPROT accession numbers. Underlined is the target MG517 in family GT2. Bootstrap values are given in each node.

The phylogenetic tree in Figure 4 shows the grouping of GT-A sequences of known structure. Different families are clustered together in each respective clade, with the exception of the proteins O53585 (GlfT of *M. tuberculosis*) and Q3J125 (a possible cellulose synthase of R. sphaeroides) that are assigned by CAZy as GT2 and are clustered in a single branch together with the only representatives of the GT13 and GT64 groups. The target sequence MG517 is located in the GT2 branch, in agreement with CAZy classification. When not refined sequence alignments were used, the MG517 sequence was always clustered in a single branch with the GT15 representative, out of the GT2 group, which further assesses the validity of the curated multiple sequence and structural alignment and the derived HMM. Note that the two GT domains of *E. coli* chondroitin polymerase 2Z86 are not assigned to individual CAZy families, but according to the phylogenetic tree, domain 1 (2Z86_1) is placed alone in a single branch whereas domain 2 (2Z86_2) lies together with the rest of GT2 proteins. 

### Structural modeling of *Mycoplasma genitalium* MG517

MG517 is a membrane-associated protein of 347 amino acid residues [13]. The Nt region (aa 1-220) shows sequence similarity with the GT-A family (Figure 2), while the Ct extension (aa 221-347) has no known homology with any other protein. Thus, we modeled the GT Nt-domain of our target MG517 protein, which includes the variable region. Different automatic modeling servers were initially tested to model MG517 structure (see Comment S1). However, the final models were strongly dependent on the server used, and didn’t allow easily selecting different templates for the variable region. Then, our approach to model the structure of the Nt-domain of MG517 was to build hybrid models by homology modeling using a combination of templates for different regions of the protein sequence.

The information contained in the clustering tree (Figure 4) allowed choosing the GT2 of *Bacteroides fragilis* as the closest homologous structure to MG517 to be used as template for the model generation. However, a first round of models using this template showed that they lost part of the consensus topology. This happens because the β-strand 7 is not resolved in the *Bacteroides fragilis* structure, and thus the models lack information for this region. Therefore, a second round of models was built, using the next closest GT2 structure according to the clustering tree, which corresponds to the second GT-A domain of *Escherichia coli* chondroitin polymerase (2Z86_2) with the whole β-strand 7 fully resolved (23% sequence identity). The three dimensional structure of the conserved region of MG517 (amino acid residues 1 to 121 and 174 to 220) was modeled using this structure as template. 

Since no consensus structure could be assigned to the GT-A variable region, our strategy to model this region (amino acid residues 122 to 173) of the target MG517 was to select different GT-A structures as templates based on the following criteria: i) one structure per GT family; ii) similar sequence length to the MG517 variable region; and iii) structures solved in complex with a ligand. Accordingly, four representative structures were used: the GT6 from *Bos taurus* (α3GalT, 1O7Q, 13% sequence identity), the GT27 from *Homo sapiens* (ppGaNAcT-2, 2FFU, 8% sequence identity), the GT2 from *Escherichia coli* (chondroitin polymerase, 2Z86, 4% sequence identity), and the GT43 from *Homo sapiens* (GlcAT-I, 3CU0, 4% sequence identity). In contrast to *de novo* modeling of this variable region, we think this approach reduces the conformational space of the variable region to geometries already identified in the GT-A fold clan. 

**Figure 5:**
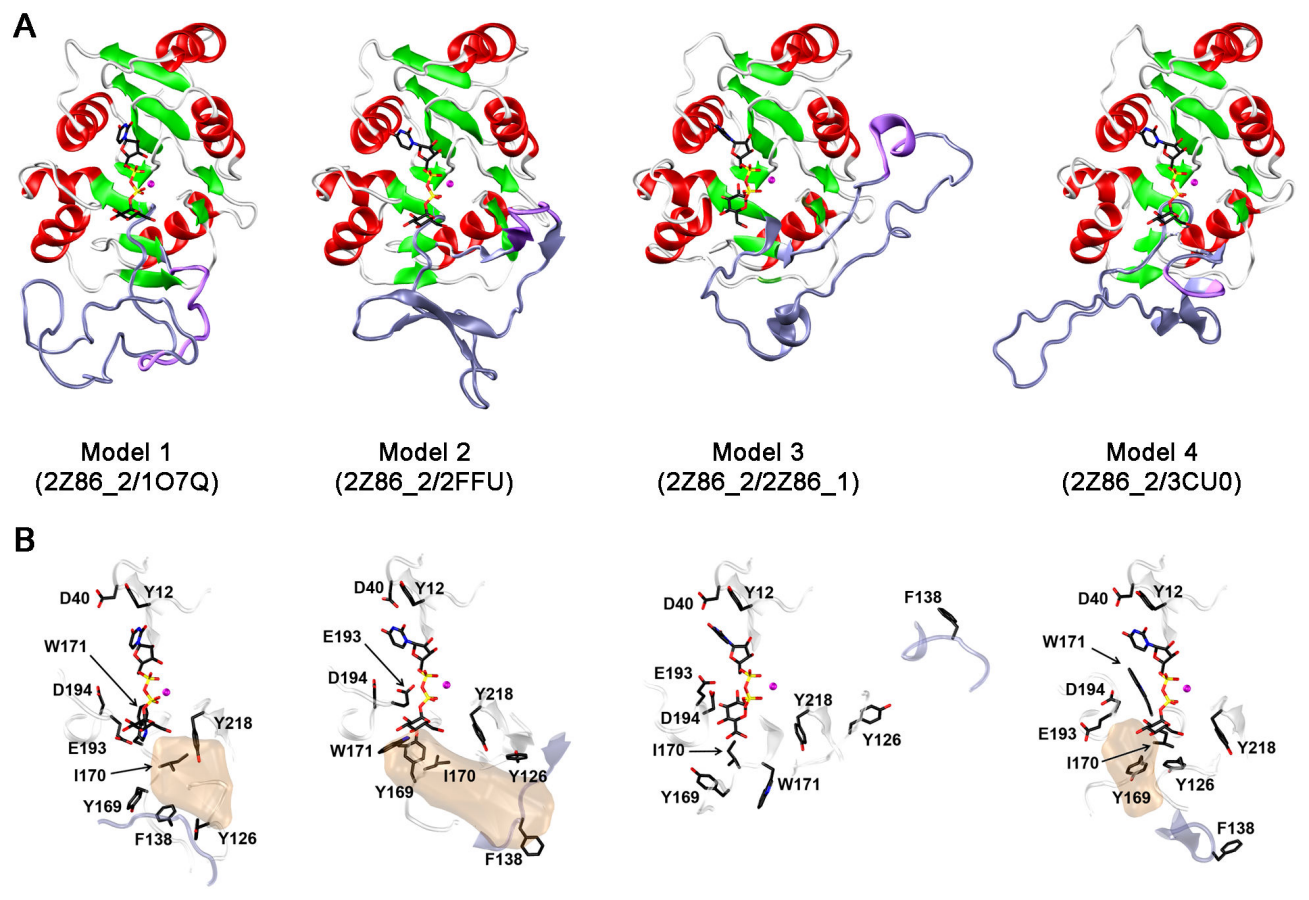
Structural models for GT MG517 (aa 1-220). A) The four models were generated using the 3D structure of *E. coli* chondroitin polymerase domain 2 (PDB 2Z86) as template for the conserved region (aa 1-121 and 174-220) and four different structures as templates for the variable region (aa 122 to 173, shown in blue). The PDB accession codes for the template structures are given in parenthesis. B) Location of the selected amino acid residues analyzed by site-directed mutagenesis (Table 2) in the four structural models. Filled volume represents the acceptor site in the original templates (except for model 3 where no ligand was present). Coordinate files of the models are available upon request.

Four different structural models of MG517 (amino acids 1 to 220) were built up from a composite of templates: 2Z86_2 for the conserved region in all models plus one of the four latter templates for the variable region in each different model. Each model also contained the ligands from the structures used as templates for the variable region (see Methods). This new round of models produced structures in which the consensus topology of GT-A is conserved in all of them (Figure 5a), with six α-helices and seven β-strands conserved in the same position of the original template. Only the β6 strand is antiparallel to the others, where the interacting β4 strand precedes always the DXD motif. Next to it there is the small β4’-strand forming a β-sheet with strands β7 and β5 in two of the models (1 and 3) and only with β7 in the other two (models 2 and 4). The variable region is located between strands β5 and β6, and each structure keeps the fold of its own template: Model 1 (1O7Q/2Z86_2) shows a large unstructured coil, Model 2 (2FFU/2Z86_2) has four β-strands, Model 3 (2Z86_1/2Z86_2) shows two α-helices out of the three seen in the template, one of them was lost during the modeling due to the sequence gap introduced in MG517 sequence as compared to the corresponding template; and Model 4 (3CU0/2Z86_2) keeps all the secondary structures from the template. Thus, at this stage no consensus structure is observed for the variable region. For the conserved region, main structural differences between models are always placed in loop regions. Furthermore, the totality of the backbone angles in these models are located in allowed regions of the Ramachandran-plot and with an average normalized DOPE Z-score of -0.3.

### Sugar-nucleotide donor binding site

The four models for the GT MG517 Nt-catalytic domain (100 structures per model, see Methods) contain the UDPGlc substrate (Figure S3). The full set of structures was used to identify the residues that are closer than 4 Å to the UDPGlc donor in each model. A list of 36 residues was obtained (Table S1), from which 9 in the conserved region were selected as potential key residues involved in binding and catalysis to be probed by site directed mutagenesis experiments. The location of these residues in each model structure is shown in Figure 5b. These residues were selected because they are conserved in the sequence alignment and/or mutations in equivalent positions in other proteins have been reported: Y12 seems to be involved in a stacking interaction with the uracil ring of UDP; D40 may stabilize the UDP by electrostatic interactions; Y126 and Y169 are the flanking residues of the variable region and are close to each other and to the Glc ring of the donor in two of the models; I170 is close to the sugar moiety of the donor; W171 is placed between the UDP and Glc rings; E193 or D194 might be the catalytic base; and Y218 substitutes a highly conserved His in GT-A enzymes. Additionally, position F138 was also considered for mutagenesis. It is located in the variable region close to the putative acceptor binding site in two of the models while it has a different orientation in the other two, thus being a probe to discriminate among models. Residues of the DXD motif [21,22] (D93, P94, D95) were not selected since their role is well known in GT-A enzymes.

### Probing active site residues by mutagenesis

**Figure 6:**
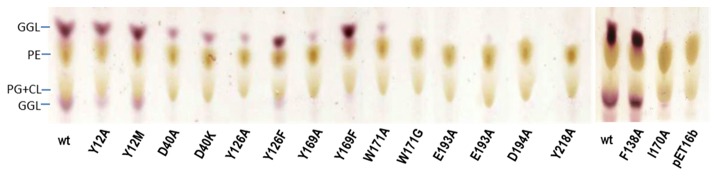
TLC analysis of lipid extracts from recombinant *E. coli* cells expressing MG517 mutants. GGL, glycoglycerolipids; PE, phosphatidylethanolamine; PG: phosphatidylglycerol; CL: cardiolipine. pET16b are control cells transformed with plasmid pET16b with no insert.

In order to choose the best representative model among the generated ones, a functional assay was performed on mutants at the selected positions. Mutants were prepared by site-directed mutagenesis, and their GT activity evaluated by two complementary assays. First, recombinant *E. coli* cells expressing each mutant protein were analyzed for glycoglycerolipid (GGL) production (*in vivo* GT assay). Since *E. coli* does not produce glycoglycerolipids, MG517 products are easily detected in total lipid extracts by TLC (Figure 6). Three groups of mutants can be observed: those with GGL formation similar to the wt enzyme (Y12A, Y12M, Y126F, F138A, and Y169F), those with reduced activity (D40A, D40K, Y126A, Y169A, and W171A), and mutants where no products are detected (I170, W171G, E193A, D194A, and Y218A). Next, the specific activities were determined in solubilized cell extracts (*in vitro* GT assay). The membrane-associated MG517 protein was extracted during cell lysis with a buffer containing CHAPS detergent and glycerol [13]. SDS-PAGE showed similar expression levels for all mutants. Activity of the solubilized extracts with UDPGal as donor and a fluorogenic ceramide derivative (Cer-NBD) as acceptor was monitored by HPLC (GT MG517 has shown to also accept ceramide in addition to the natural diacylglycerol acceptor, which is convenient for activity assays using a fluorescent-labeled ceramide derivative). Results are summarized in Table 2.

**Table 2:** Enzymatic activity of GT MG517 mutants.

Mutant	Vo/[prot] (µM/mg.min)	activity (%)
MG517 wt	14.5	100
Y12A	7.05	49
Y12M	8.91	61
D40A	0.17	1,2
D40K	0.25	1,7
Y126A	2.9	20
Y126F	5.55	38
F138A	15.9	109
Y169A	2.56	18
Y169F	54.0	372
I170A	0.32	2.2
W171A	6.25	43
W171G	0.02	0,13
E193A	0	0.00
D194A	0.0035	0.02
Y218A	0.47	3.2

Total protein concentration was determined by the BCA assay. Activity was determined at 1 mM UDPGal, 100 µM Cer-NBD solubilized with BSA (25µM), in 10 mM HEPES pH 8.0, 10 mM CHAPS, 10% glycerol), 10 mM MgCl_2_ at 25°C. Specific activity under these conditions (v_0_/[prot]) was expressed as the initial rate of product formation (v_0_ (µM·min^-1^)) per milligram of total protein in the extract.

The role of the mutated residues is analyzed based on the structural location in our models and compared to other GT-A enzymes:

Tyr12 is found well conserved in the sequence alignment with 33% identity among GT-A enzymes with solved 3D structures (Figure 2). It is part of a hydrophobic pocked formed by residues at the end of the β1-strand that accommodates the uracil ring of the UDPGlc donor. For instance, *Mycobacterium* MAP2569c and GpgS (PDB entries 3CKJ and 3E26 in Table 1) [20,23] have a Leu (L57 and L52, respectively) in the equivalent structural position that stacks with the uracil ring. Mutants Y12A and Y12M retain 49% and 61% of the wt MG517 activity, indicating that it is not an essential residue and that the possible staking interaction (more clearly seen in Model 3) is not critical for activity.

Asp40 is another well conserved residue with 50% identity. It is part of the tetrad of aspartates proposed as recognition and catalytic elements in families GT2, 7, 13, and 43, based on the X-ray structure of SpsA (D39-D98-D99-D191) (PDB entry 1QG8 in Table 1) [24]. Asp 40 at the end of strand β2 is equivalent to Asp39 in SpsA, which coordinates N-3 of the uracil base. Mutants D40A and D40K have strongly reduced activity (<2% than the wt MG517), consistent with the proposed role. However, similar mutations in other proteins have different effects: mutant D44A in ExoM from *Sinorhizobium meliloti* also results in the loss of activity [25], as well as D156Q in murine ppGaNTase-T1 (PDB entry 1XHB in Table 1) which retains only 0.1% of wt activity [26], whereas mutant D41A in *Salmonella* WbbE does not appear to be critical for activity [22].

Tyr126 is placed at the end of strand β5, just at the beginning of the variable region, and close to the Glc unit of the UDPGlc donor in Models 1 and 4 for which the Cα carbon is close (≈9Å) to the Cα-carbon of Tyr169 at the end of the variable region. Mutants Y126A and Y126F retain 20% and 38% of wt MG517 activity, suggesting the involvement of Tyr126 in substrate binding. In the same region at the end of strand β5, α3GalT (PDB entry 1O7Q in Table 1) has Gln247 which forms multiple interactions with acceptor substrates, and the Q247E mutation reduces significantly the transferase activity [27]. 

Phe138 is in the variable region, with the side chain close to the putative acceptor site in Models 1 and 2. Mutation to Ala (F138A) has no effect on activity, indicating that this residue does not interact with the substrates. Therefore, models 1 and 2 seem to be less appropriate than models 3 and 4 to describe the variable region.

Tyr169, Ile170, and Trp171 are located at the beginning of strand β6 after the variable region but are not conserved residues. Tyr169 is close to Tyr126 at the beginning of the variable region in Models 1 and 4, both in the acceptor binding site (see above). Mutant Y169A retains 18% of activity consistent with this role. Surprisingly, Y169F results in a more active protein, with 380% activity than the wt MG517. Moreover, this mutant shows a different products profile (in the *in vitro* activity assay), producing mainly the monoglycosylated product (MGDAG) and essentially none of the diglycosylated one (DGDAG). A tentative interpretation is that removal of the OH group of Tyr169 increases the hydrophobicity of the acceptor binding site favoring binding of the lipid acceptor and disfavoring the binding of the first glycosylated product that would place a more polar Glc residue in the same position for the second glycosyl transfer. This mutant will deserve further analysis to characterize its biochemical properties. 

Ile170 is in the region that aligns with H280 in α3GalT [27], and with positions 266 and 268 in Histo-blood group ABO system transferases (i.e.PDB entry 3IOH in Table 1), known as key residues in defining donor specificity for Gal or GalNAc sugar nucleotides [28]. The mutant I170A in MG517 has a strongly reduced activity (<2% than that of the wt enzyme) consistent with its direct role in donor specificity.

Trp171 is conserved in about 20% of the GT-A sequences (6158 sequences from the CAZy data base aligned with our HMM profile) while there is mainly a Gly residue in the others. The crystalized GT-A enzymes (Table 1) belong to this second group, where near 70% of the structures have a Gly, and no one has a Trp in this position. Mutant W171G in MG517 only retains 0.1% of wt activity. This result seems to indicate that these two GT-A groups may have evolved separately to accommodate either a Trp or a Gly that are not interchangeable in that position. Interestingly mutant W171A has 43% activity, where a larger side chain than Gly recovers part of the activity. 

Glu193 or Asp194 are the candidates to act as general base in the catalytic mechanism. One of them may be part of the proposed tetrad of Asp proposed as recognition and catalytic elements [22,24] together with Asp40, Asp93, and Asp95 (the last two as the DXD motif) in MG517. Mutant D194A still retains detectable activity (0.02%) whereas E193A is fully inactive (confirmed by activity assays with purified enzyme at high concentration), so Glu193 is the candidate to act as general base to deprotonate the hydroxyl acceptor in the GT MG517 mechanism.

Finally, Y218 is located in a loop next to strand β7 at the end of the conserved GT-A domain. This residue aligns with H258 in GpgS which corresponds to a highly conserved His in GT-A enzymes playing an important role in metal binding [20,23,29]. Indeed, mutant Y218A retains only 3% of wt activity suggesting that Tyr218 might coordinate the divalent cation together with D95 and the two phosphate moieties of UDP.

In conclusion, the mutational analysis at these selected residues allows choosing the best representative models. Glu193 is proposed as the catalytic residue in line with Model 3, while the rest of protein-ligand interactions in the active site are properly described by Model 4. Mutations at Asp40, Tyr126, Tyr169, Ile170 and Tyr218, which define the glycosyl donor binding site in Model 4, notably alter enzymatic activity. 

### Model refinement by long scale Molecular Dynamics

**Figure 7:**
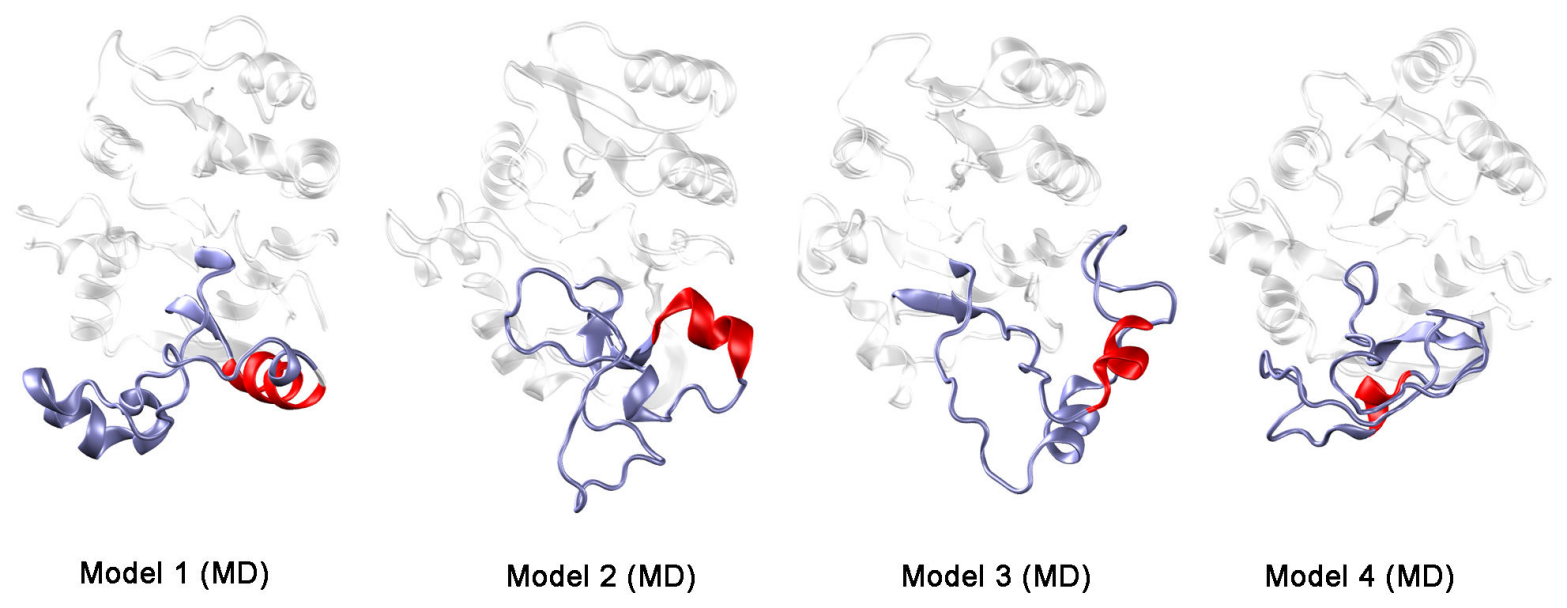
MG517 structural models after MD simulations. The variable region is highlighted, with the converged α-helix in red.

The four structural models of MG517 generated by homology modeling with hybrid templates keep the consensus topology of GT-A proteins (Figure 5). However, we could not assign any consensus structure to the variable region, as it was largely influenced by the chosen template. A series of long molecular dynamics (MD) simulations (one microsecond each, Table S2) were performed on each model attempting at recovering part of the natural structure in the variable region of MG517. Given the artifacts that the low sequence identity between the variable region of MG517 and the corresponding templates could have introduced in the structure of MG517, only the best structure in terms of DOPE score and backbone angles distribution was chosen for each round of models to perform long MD simulations. After 600-850 ns, the simulations were stable: the RMS deviations of backbone atoms were average 3.5 Å (Table S3) and no more changes in secondary structure were detected (Figure S4). At the end of the MD simulations, the four hybrid structures kept the global fold and essentially, all the secondary structure elements of the conserved region obtained by the previous homology modeling (Figure 7). No important conformational changes have been detected in the conserved region. 

Regarding the variable region, the four independent long MD simulations have undergone important conformational changes and no common global fold was found for it, except for a common α-helix at the beginning of the variable region (see Movies S1, S2, S3 and S4, comparing the model structures before and after MD simulations, for models 1 to 4 respectively). It is important to notice that such an α-helix was not present in the starting point structures of the MD simulations for three out of the four initial models. Indeed, the variable regions of Models 2 and 4 were mainly formed by β-sheets. At the end of the MD simulations (Figure 7), the first two β-strands have refolded into an α-helix in both models (located in the same position) whereas the remaining β-strands have unfolded. Likewise, the variable region of Model 1 had just a small 3_10_ helix of 3 residues long. This helix is not only kept, but it is extended during the MD simulation by refolding part of the subsequent coil. Finally, the variable region of Model 3 showed two α-helices and no β-sheets. At the end of the simulation, both helixes are maintained, although they changed their spatial position. Compared to the other models, the starting position of the first α-helix in this model is shifted around 5 amino acids.

In conclusion, long-scale MD simulations have allowed merging all structural information coming from the four models into a unified topology, in which the conserved region of MG517 keeps the GT-A fold, whereas the beginning of the variable region is presumably formed by a 10 aminoacids long α-helix. However, the exact spatial orientation of this helix and the rest of the structure of the variable region, still remain elusive at this modeling stage. After MD simulations, it is confirmed that models 3 and 4 best describe the mutational results reported above.

### Diacylglycerol acceptor binding site

Three out of the four GT-A structural templates used to generate our MG517 models include also their respective acceptor molecules in the structure (GalNAcβ4Glc in *Bos Taurus* 1O7Q, an octapeptide in *Homo sapiens* 2FFU, and Galβ3Gal(6-SO_4_) in *Homo sapiens* 3CU0). Interestingly, all acceptor molecules bind in the structurally variable region of each template, supporting the idea that the structural and sequential diversity in this region is responsible of the acceptor substrate specificity. In order to confirm this hypothesis in the framework of MG517, the preferential location of the natural acceptor diacylglycerol (DAG) in this GT enzyme was predicted by docking.

DAG is preferentially placed in the variable region (Figure S5 shows the accessible volume of DAG onto MG517 modeled structures). The location of the DAG molecule along the MG517 structure is similar to the position of the natural acceptor substrates in their respective GT-A structures used as templates being the relative interaction energies within the same range (Table 3). The DAG molecule was also docked to the template structures, but no binding was detected. These results reinforce the idea that the structural and sequential diversity in the variable region of GT-A proteins dictates the substrate specificity for different ligands. 

**Table 3:** Estimation of interaction energies of the acceptor substrates to modeled and template structures.

Structure	interaction energy (kcal/mol) **^a^** original acceptor **^b^**	interaction energy (kcal/mol) **^a^** DAG acceptor **^c^**
Model 1 (2Z86_2/1O7Q)		**-5.3 / -5.0**
Template 1O7Q	-6,5	n.d.
Model 2 (2Z86_2/2FFU)		**-5.8 / -5.4**
Template 2FFU	-5.3	n.d.
Model 3 (2Z86_2/2Z86_1)		**-5.8 / -5.4**
Template 2Z86_1	---	n.d.
Model 4 (2Z86_2/3CU0)		**-5.7 / -5.4**
Template 3CU0	-5.6	n.d.

a estimated interaction energies calculated by Autodock for the most probable binding events. The intervals of estimated energies are given.

b estimated interaction energies for the original acceptor in its original X-ray structure used for modeling the variable region. Ligands were: GalNAcβ4Glc in 1O7Q, an octapeptide in 2FFU, and Galβ3Gal(6-SO4) in 3CU0. No ligand present in 2Z86_1.

c estimated interaction energies for the DAG acceptor (dipropionylglycerol) in the modeled structure. DAG binding is not detected (n.d.) in the original X-ray structures used as template for the variable region.

## Conclusions

We have presented here a three-dimensional model of an essential gene product of *Mycoplasma genitalium*, the MG517 glycosyltransferase that synthesizes mono- and di-glycosyldiacylglycerols. Four different models were built by combining structural information from different GT-A structures. *In vitro* measurements of selected MG517 mutants, designed on the basis of the structural models, allows *a posteriori* to choose the best representative models. Glu193 is proposed as the catalytic base in line with Model 3, while the rest of protein-ligand interactions in the active site are properly described by Model 4. Mutations at Asp40, Tyr126, Tyr169, Ile170 and Tyr218, which define the glycosyl donor binding site in Model 4, notably alter enzymatic activity. 

Our models also shed some light on the lipid acceptor binding site, which is putatively located along the variable region. In our opinion, the structural and sequential diversity in this region in different GT-A enzymes is responsible of the acceptor specificity. The promiscuity of MG517 enzyme to accept both non-glycosylated and mono-glycosylated lipids may be controlled in part by the non-conserved Tyr169 residue. However, the exact geometry of the acceptor binding site, in the variable region, cannot be described at this stage of the models. We propose that an α-helix is formed at the beginning of the variable region, and that an important hydrophobic patch is exposed, which is compatible with protein dimerization as suggested for other GT-As, with protein-membrane association to facilitate lipid ligand binding, or even with binding to the C-terminus domain of the protein, which is missing in our models.

We have established the consensus sequential and structural topology of the GT-A family of enzymes (Figure 2 and Figure 3), which to the best of our knowledge was not studied in such detail before. The curated refinement of multiple sequence alignments, which incorporate structural information, led to a Hidden Markov Model profile representative of the GT-A fold clan. This new profile has many potential applications such as the detection of homologous sequences in recently sequenced genomes, or to guide the alignment of GT-A sequences. The modeling protocol we have here applied should be taken into consideration when attempting to model new GT-A structures because of the lack of clear homologous structures to be used as template for the intrinsically variable region in this family of proteins.

In summary, by a combination of *in silico* structure modeling and *in vitro* measurements, we have defined important structure-function relationships in a GT-A glycolipid synthase. The model needs further refinement, in particular incorporation of the non-conserved C-terminal region that will complete the acceptor binding site and probably define the region interacting with the membrane. While attempts to solve the structure by X-ray crystallography are not yet successful, the structure-function relationships that emerge from the models here reported are the first insight to define enzyme-ligands specificity with the goal of designing specific inhibitors to this and other related GT2 glycolipid synthases addressed to discover new treatments against mycoplasma infections based on a novel target.

## Methods

### Data retrieval

The list of family GT-A enzymes characterized so far was accessed at CAZy database [9]. The three dimensional structures of these enzymes were downloaded from the Protein Data Bank (PDB) and their corresponding amino acid sequences from UniProt (Table 1).

### Structural and Sequence alignment

GT-A three dimensional structures were superimposed with POSA server [30]. Secondary structure annotations from known three dimensional protein structures were obtained with DSSP [31]. Secondary structure predictions for unknown protein structures were performed with PSI-PRED server [32]. Protein sequences were extracted from the PDB files and then aligned with PROMALS server [33] which implements a profile-based multiple sequence alignment algorithm that incorporates secondary structure information. Curation of the multiple sequence alignment was done by visually inspecting the superimposed structures with VMD software [34]: those amino acids of different structures located in the same region of the space were manually placed in the same column in the alignment; the secondary structure annotation was also used to guide the curation of the alignment. HMMER software [35] was used to build a Hidden Markov Model (HMM) profile for the GT-A fold clan with the curated alignment. A new multiple sequence alignment, with the complete proteins sequences from UniProt, was generated using this HMM profile. Clustering of the sequences in the HMM alignment was performed by means of the Neighbor-joining algorithm [36] using a BLOSUM62 scoring matrix as implemented in PHYLIP [37]. Consistency of the generated tree was assessed by a bootstrap resampling of 10000 datasets. The booststrap was performed as the final step of the CONSENSUS program, which draws the consensus tree, and the final values assigned to each node of the tree.

### Homology modeling

The structural models of *Mycoplasma genitalium* glycosyltransferase MG517 (N-t domain, aa 1-220) were built by means of comparative homology modeling using MODELLER v.9.8 [38]. Multiple GT-A structures were used as templates. Four different series of models were built using a combination of the second GT2 domain of the chondroitin polymerase structure from *E. coli* (2Z86 PDB code: chain amino acids 430 to 632) for the conserved region plus one of the following four templates for the structurally variable region: i) the bovine GT6 galactosyltransferase (α3GalT) structure (1O7Q PDB code: chain amino acids 242 to 287); ii) the human GT27 polypeptide N-acetylgalactosaminyltransferase 2 (ppGaNTase-T1) structure (2FFU PDB code: chain amino acids 247 to 314); iii) the other GT2 domain of the chondroitin polymerase structure from *E. coli* (2Z86 PDB code: chain amino acids 263 to 335); and iv) the human GT43 β-glucuronosyltransferase 3 (GlcAT-I) structure (3CU0 PDB code: chain amino acids 213 to 259). The multiple sequence alignment obtained previously was used to guide the modeling of MG517 structure on the basis of the above mentioned templates. 10 different structural models were generated for each combination of templates, starting from different randomized coordinates. Each model was refined with a short simulated annealing protocol as implemented in MODELLER. For each of these models, 10 additional loop refinement models were generated for the portion of structure without alignment to the templates, as implemented in MODELLER [39]. The models included also the donor substrate (UDPGlc) in the equivalent position of the UDP molecule in the respective templates. The full UDPGlc unit was modeled from the UDP-glucuronic acid present in the 2Z86 template. The final 100 structure models generated for each combination of templates were assessed by means of empirical scoring energies such as the DOPE score [40] and by analyzing the distribution of Ramachandran dihedral angles with PROCHECK [41]. 

### Molecular dynamics simulations

One representative MG517 structure of each of the four series of models, to be used for starting the molecular dynamics simulations, was chosen among the top 20 DOPE scoring structures of each series of models using the following criteria (some of them based on bibliography and sequence conservation): i) amino acids D40, D93, D95, D194 should not be placed farther than 6 Å of ligand UDPGlc in the selected model, and ii) amino acid E193 or D194 should be oriented towards the scissile bond between the diphosphate and glucose moieties in UDPGlc.

By means of software GROMACs v4.5.3 [42], a long molecular dynamics simulation (MD) was applied to each of the four structures selected previously. The protonation state of each initial structure was assigned by means of the server H++ [43]. The UDPGlc ligand was incorporated to the structures in template equivalent positions. The simulations were performed with an Amber force field, cubic box, solvent explicit treatment and neutral charge of the system, adding ions to neutralize the system at a final concentration of 0.15 M. Amber parameters for the ligand were taken from [44]. The molecular dynamics simulation was extended up to one microsecond for each one of the four structures. At the end of each MD simulation, all the generated structures during the trajectory were grouped into clusters, using the gromos clustering method and RMSD as a metric [45]. A consensus between the cluster size (the biggest), the temporal location (the closest to the microsecond), and the ergodicity behavior is used to choose the representative structure of each MD simulation.

### Acceptor Binding Site

Autodock v.4.2.3 [46] was used to predict the putative binding sites between diacylglycerol substrate (acceptor) and MG517 protein structures. The protein structure was taken from the resulting MD models obtained as described in the previous section. A blind docking strategy was followed in which the whole protein structure was scanned for putative binding sites. Dipropionylglycerol was used as the acceptor (DAG) substrate probe. We computed solvation, electrostatic and affinity grid potentials on the whole protein structure for each atom type in the substrate, by means of Autogrid v.4.2.3 [46]. The single bonds of the substrate molecule were considered flexible during the docking search. 100 rounds of a genetic algorithm were performed for docking. For each round, an initial population of 300 members was considered, with randomized initial position and orientation coordinates, and randomized conformations of the substrate flexible bonds. The genetic algorithm was extended up to 27000 offspring generations, with a maximum of 25000000 energy evaluations.

### MG517 mutants by side directed mutagenesis

Single point mutations at selected residues (Y12A, Y12M, D40A, D40K, Y126A, Y126F, F138A, Y169A, Y169F, I170A, W171A, W171G, E193A, D194A and Y218A) were prepared by PCR-SDM following a modified QuickChange protocol using extended primers [47]. pET44b-*mg517*, a plasmid encoding for GT MG517 [13] was used as template for mutagenesis using the primers listed in Table S4. Competent *E. coli* DH5α cells were transformed with the mutagenesis reactions after DpnI digestion, and positive transformants were sequenced. 

### Expression of GT MG517

*E. coli* BL21(DE3Star) cells were co-transformed with pET44b-*mg517* (wt and mutants), and pGro7 (from Takara Bio Ltd), a plasmid encoding for *E. coli* chaperones GroEL and GroES under the control of an *araB* promoter and containing a chloramphenicol-resistance gene. Cells were grown in LB medium containing ampicillin (100 μg/mL) and chloramphenicol (25 μg/mL) at 37°C. When the optical density of the culture reached 0.3, expression of chaperones was induced by adding L-arabinose (2 g/L). The culture was further incubated for 30 min at 37 °C before IPTG (1 mM) induction of MG517 expression. Cells continued to be grown for 16 h at 25 °C before harvesting.

### Lipid extraction and TLC analysis

The cellular pellet of recombinant of *E. coli* BL21(DE3) expressing MG517 was subjected to lipids extraction with chloroform/methanol 2:1 (v/v). The organic fraction was concentrated by solvent evaporation under a steam of nitrogen, and analyzed by TLC (silica gel plates) developed with chloroform/methanol/water 65:35:4 (v/v), and stained with sulfuric/methanol/water (45:45:10 v/v) for visualization. 

### Specific activity

Cell-free extracts of solubilized MG517 were prepared as previously reported for the wt enzyme [13]. Cells from 4 mL cultures were harvested by centrifugation (6000xg for 10 min at 4°C), washed with 2 mL 0.9% NaCl, resuspended in 1 mL of extraction buffer (20 mM CHAPS, 20% glycerol, 20 mM HEPES pH 8.0, 20 mM MgCl_2_), and lysed by sonication. After centrifugation (16000xg for 15 min at 4 °C), the soluble fraction contained MG517 together with membrane lipids required for activity. Total protein concentration was determined by the BCA (bicinchoninic acid) assay (Pierce).

Specific activity was determined with UDPGal as donor and Ceramide-NBD (N-[6-[(7-nitro-2-1,3-benzoxadiazol-4-yl)amino]hexanoyl]-D-erythro-sphingosine) as acceptor substrates. Protein extracts (60 µg total protein in 50 µL extraction buffer), were mixed with the acceptor substrate solubilized with BSA, and incubated for 30 min at 4°C. Then, the reaction was started by adding the donor substrate and incubated at 25°C. Final reaction conditions were: 1 mM UDPGal, 100 µM ceramide-NBD, 25 µM BSA, 10 mM CHAPS, 10% glycerol, 10 mM HEPES pH 8.0, 10 mM MgCl_2_ in a final reaction volume of 100 µL. Aliquots of 10 μL were withdrawn at different times (1 to 30 min) and mixed with 40 μL MeOH to stop the reaction. After centrifugation (16000xg for 10 min) to remove any traces of precipitated proteins, samples were analyzed by HPLC (Agilent 1200) with a fluorescence detector (λ_ex_ 470 nm, λ_em_ 530 nm): 10 μL injection, Novapack C18 reverse-phase column eluted with acetonitrile/H_2_O (3:1) at 1 mL/min flow rate. 

## Supporting Information

Comment S1 **Initial attemps of automatic modeling.**
(PDF)

Figure S1 **Sequence and secondary structure alignment of GT-A domains with 3D structures solved by X-ray crystallography.** MG517 sequence and predicted structure are marked by a black arrow on top of sequence and structural alignment. Mutated residues pointed by orange arrows.
(TIF)

Figure S2 **Structural superimposition of the GT-A domain (excluding the variable region) of glycosyltransferases with known 3D structure.** Helix 1, 2 and 3 are colored in red, helix 4, 5 and 6 in orange. β-Strands from yellow to green, DXD motif as purple turn in the middle of the picture..
(TIF)

Figure S3 **Sugar nucleotide binding site in the modeled structures.** UDP-Glc is at the center of the picture. Mg^2+^ is shown as a green ball. Neutral polar residues in green, negative residues in red, hydrophobic residues in grey, rest of protein as white color surface..
(TIF)

Figure S4 **MD trajectories showing the evolution of secondary structure elements.** DSSP colors are used. GT-A consensus secondary structure (Figure S1) is shown on the right of the picture.
(TIF)

Figure S5 **Docking of dipropionylglycerol to the four structural models after MD simulations.** The most energetically favored positions are showed in red, all of them close to the variable region (marked in blue in the structures). Model 1 (-5.3/-5 kCal/mol), Model 2 (-5.8/-5.4 kCal/mol), Model 3 (-5.8/-5.4 kCal/mol), Model 4 (-5.7/-5.4 kCal/mol).
(TIF)

Table S1 **Residues of GT MG517 (Nt GT domain, aa 1-220) located at <4Å from the UDPGlc donor in the four structural models.** The number of structures out of 100 structures generated per model (see Material and Methods) are given. Highlighted residues are those selected for mutagenesis experiments.
(PDF)

Table S2 **Summary of MD simulations**
(PDF)

Table S3 **Mean RMSD and RMS fluctuations (in parenthesis) for the full structure and selected amino acid residues.** Donor movement during the MD simulations.
(PDF)

Table S4 **Primers used for SDM-PCR.**
(PDF)

File S1 **Hidden Markov Model (HMM) profile of the GT-A fold as ASCII file (hmmer format).**
(TXT)

Movie S1 **3D structure of MG517 Model 1 (top) and Model 1 (MD) after molecular dynamics (bottom) rotating on the Y-axis.**
(MPG)

Movie S2 **3D structure of MG517 Model 2 (top) and Model 2 (MD) after molecular dynamics (bottom) rotating on the Y-axis.**
(MPG)

Movie S3 **3D structure of MG517 Model 3 (top) and Model 3 (MD) after molecular dynamics (bottom) rotating on the Y-axis.**
(MPG)

Movie S4 **3D structure of MG517 Model 4 (top) and Model 4 (MD) after molecular dynamics (bottom) rotating on the Y-axis.**
(MPG)
