# Supplementary material for: Structure-Function Features of a Mycoplasma Glycolipid Synthase Derived from Structural Data Integration, Molecular Simulations, and Mutational Analysis
Source: PLoS One. 2013 Dec 3;8(12):e81990. doi: 10.1371/journal.pone.0081990 (PMC3849446; doi:10.1371/journal.pone.0081990)
Supplement: Comment S1 — Initial attemps of automatic modeling. (PDF) [file pone.0081990.s001.pdf]

Comment S1. Initial attemps of automatic modeling were performed by means of servers HHPred (http://toolkit.tuebingen.mpg.de/hhpred), I-TASSER (http://zhanglab.ccmb.med.umich.edu/I-TASSER/), and LOMETS (http://zhanglab.ccmb.med.umich.edu/LOMETS/). The table shows the results by each server. The strong dependence of the models to the templates regarding the variable region, and the limitations to select different templates for different regions led us to discard this strategy.

**Servers**	**HHPRED**	**I-TASSER**	**LOMETS**
Template[a]	2Z86/3BCV/1QG8	3BCV/1XHB/2D7I/2Z86	2Z86
Selection[b]	Optimal templates	Automatic	Automatic
Database[c]	PDB	Non redundant (automatic)	Non redundant (automatic)
Model[d]	Merged structures	Threading/Fragments combined	Threading

[a] Templates selected by each server to perform the model.
[b] Templates selection. Only HHPRED allows some kind of template selection: Automatic optimal templates found or "by user". I-TASSER and LOMETS do not allow template selection.
[c] Database used for template selection. Only HHPRED allows to select the database.
[d] Method to build the modeled structure. HHPRED merges the templates if various are selected. I-TASSER combines different fragments from different templates modeled by threading.
