# Supplementary figures and images for: Structure-Function Features of a Mycoplasma Glycolipid Synthase Derived from Structural Data Integration, Molecular Simulations, and Mutational Analysis
Source: PLoS One. 2013 Dec 3;8(12):e81990. doi: 10.1371/journal.pone.0081990 (PMC3849446; doi:10.1371/journal.pone.0081990)

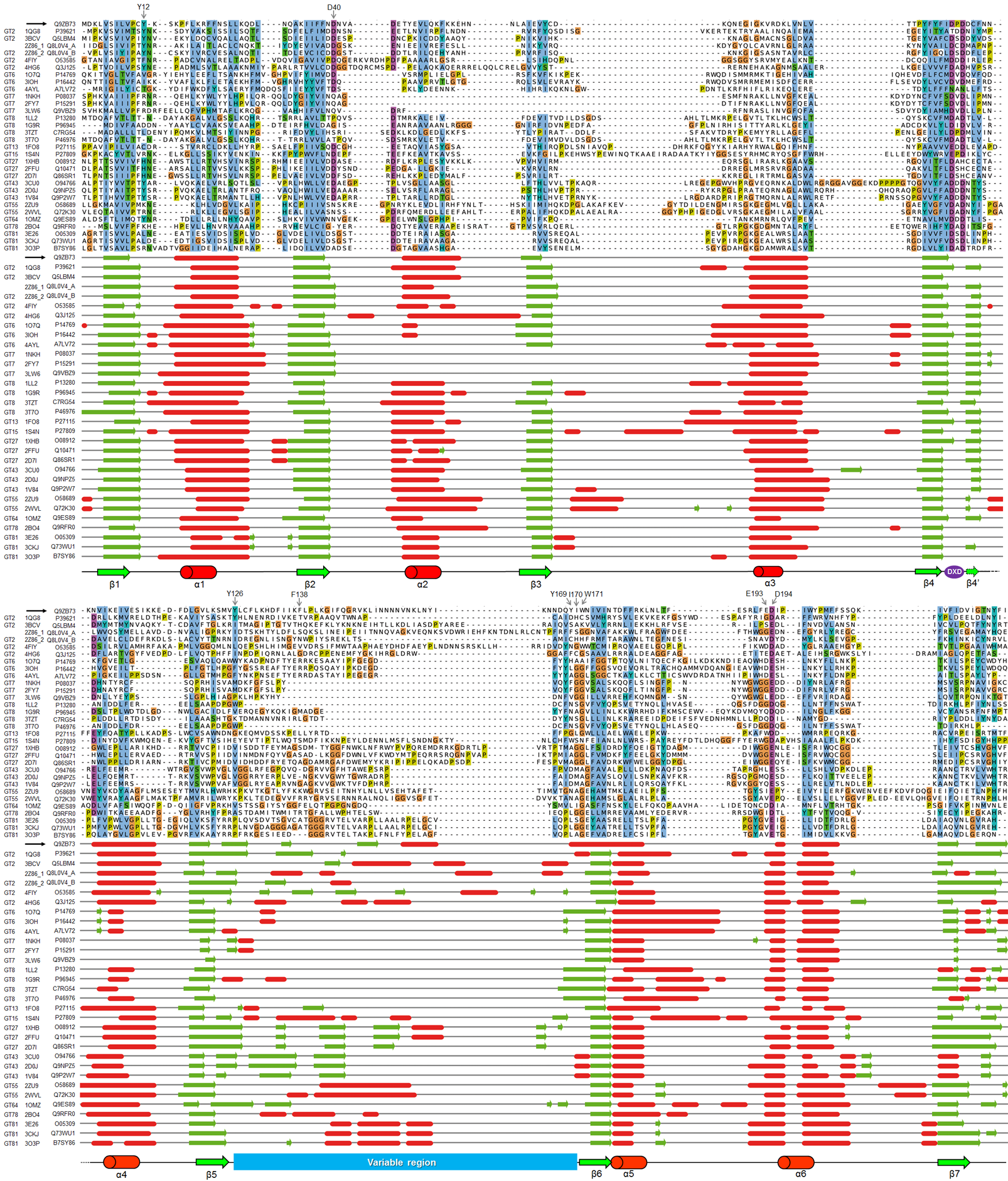

Supplement: Figure S1 — Sequence and secondary structure alignment of GT-A domains with 3D structures solved by X-ray crystallography. MG517 sequence and predicted structure are marked by a black arrow on top of sequence and structural alignment. Mutated residues pointed by orange arrows. (TIF) [file pone.0081990.s002.tif]

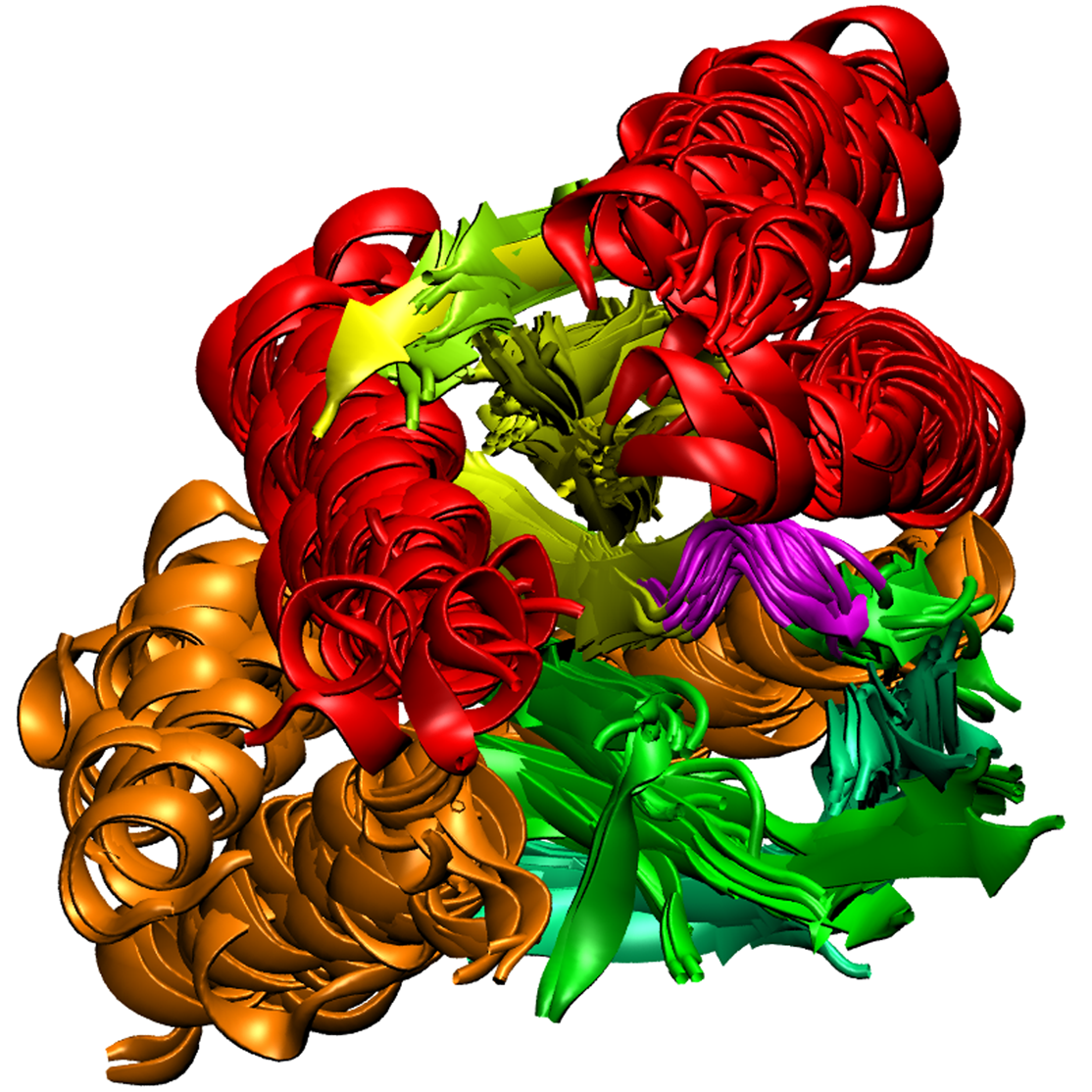

Supplement: Figure S2 — Structural superimposition of the GT-A domain (excluding the variable region) of glycosyltransferases with known 3D structure. Helix 1, 2 and 3 are colored in red, helix 4, 5 and 6 in orange. β-Strands from yellow to green, DXD motif as purple turn in the middle of the picture.. (TIF) [file pone.0081990.s003.tif]

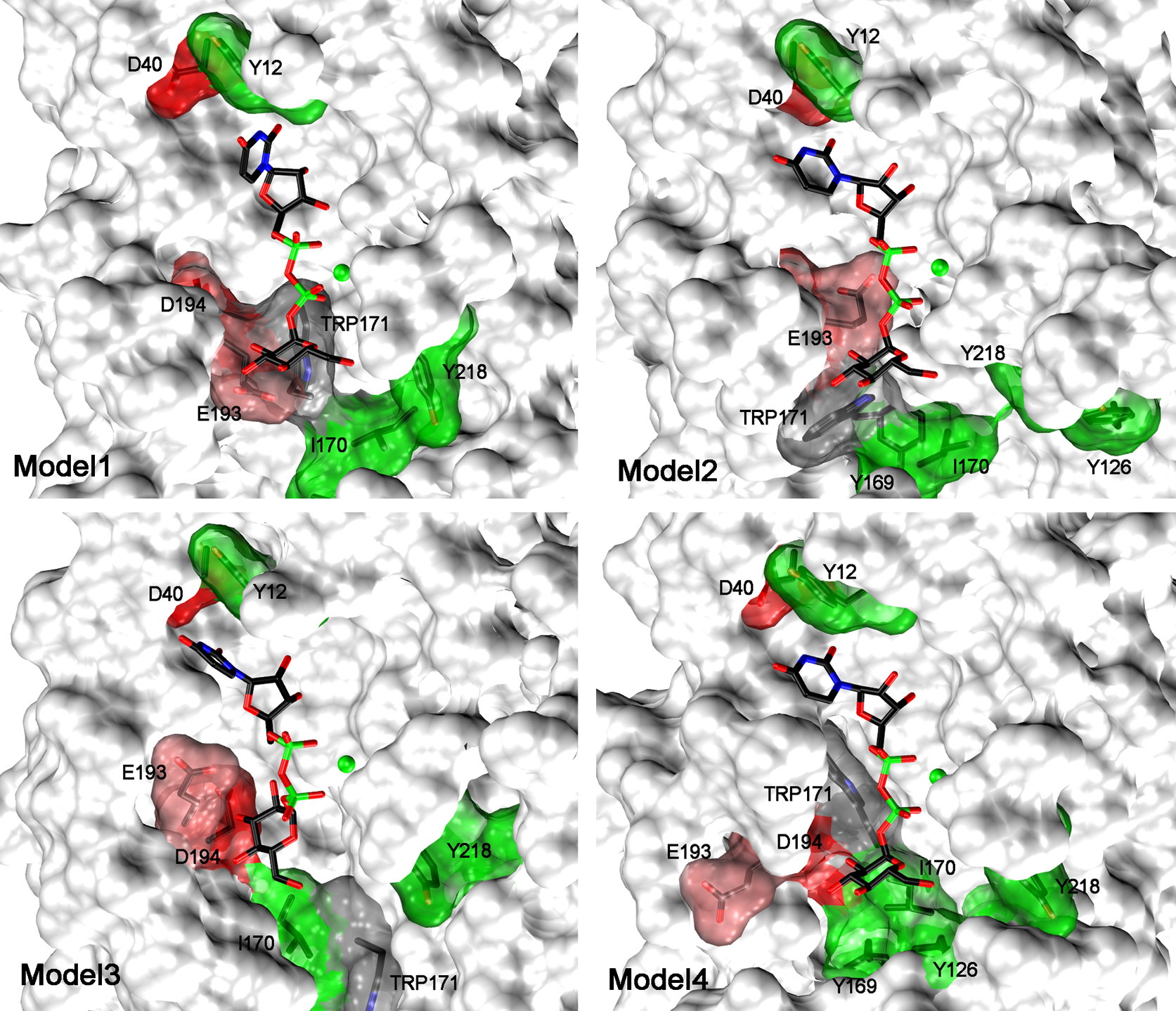

Supplement: Figure S3 — Sugar nucleotide binding site in the modeled structures. UDP-Glc is at the center of the picture. Mg2+ is shown as a green ball. Neutral polar residues in green, negative residues in red, hydrophobic residues in grey, rest of protein as white color surface.. (TIF) [file pone.0081990.s004.tif]

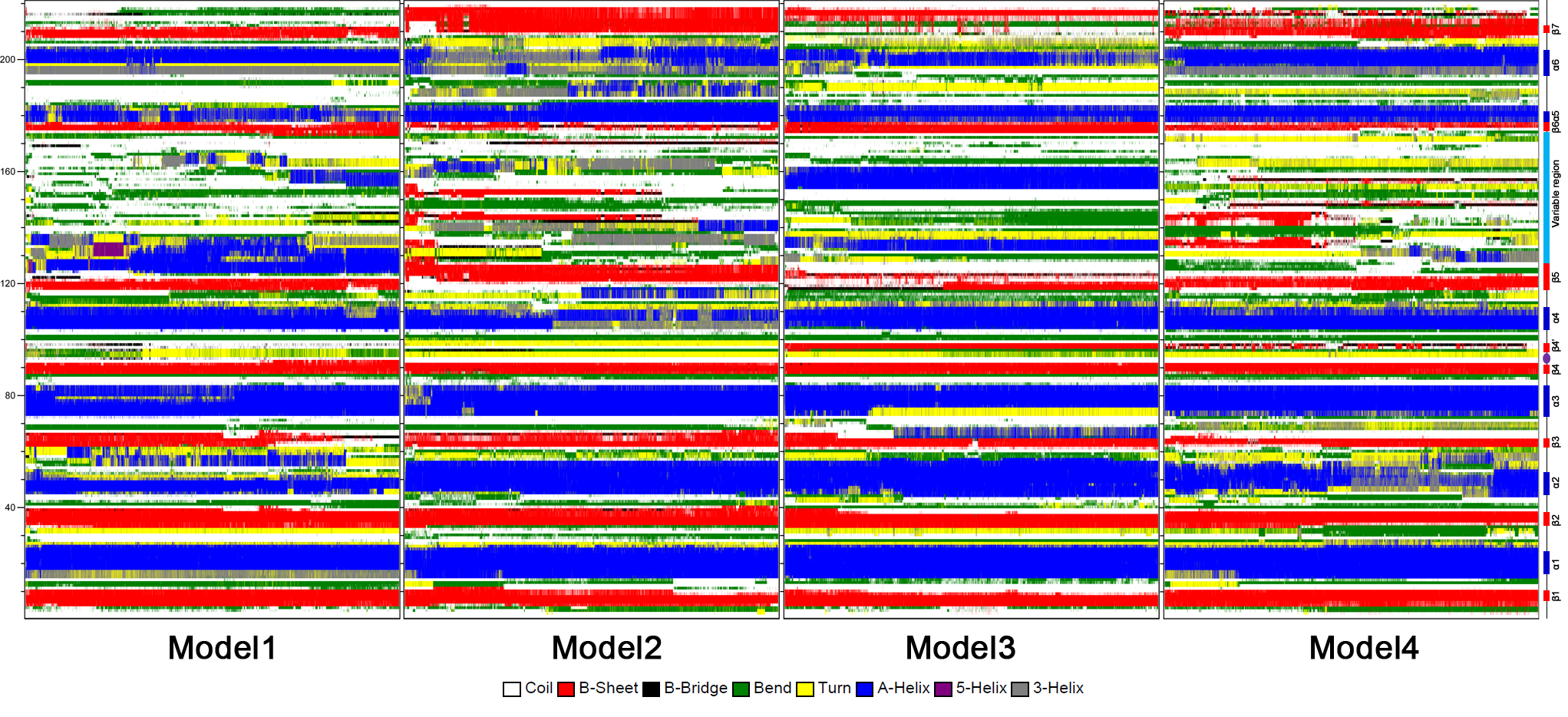

Supplement: Figure S4 — MD trajectories showing the evolution of secondary structure elements. DSSP colors are used. GT-A consensus secondary structure (Figure S1) is shown on the right of the picture. (TIF) [file pone.0081990.s005.tif]

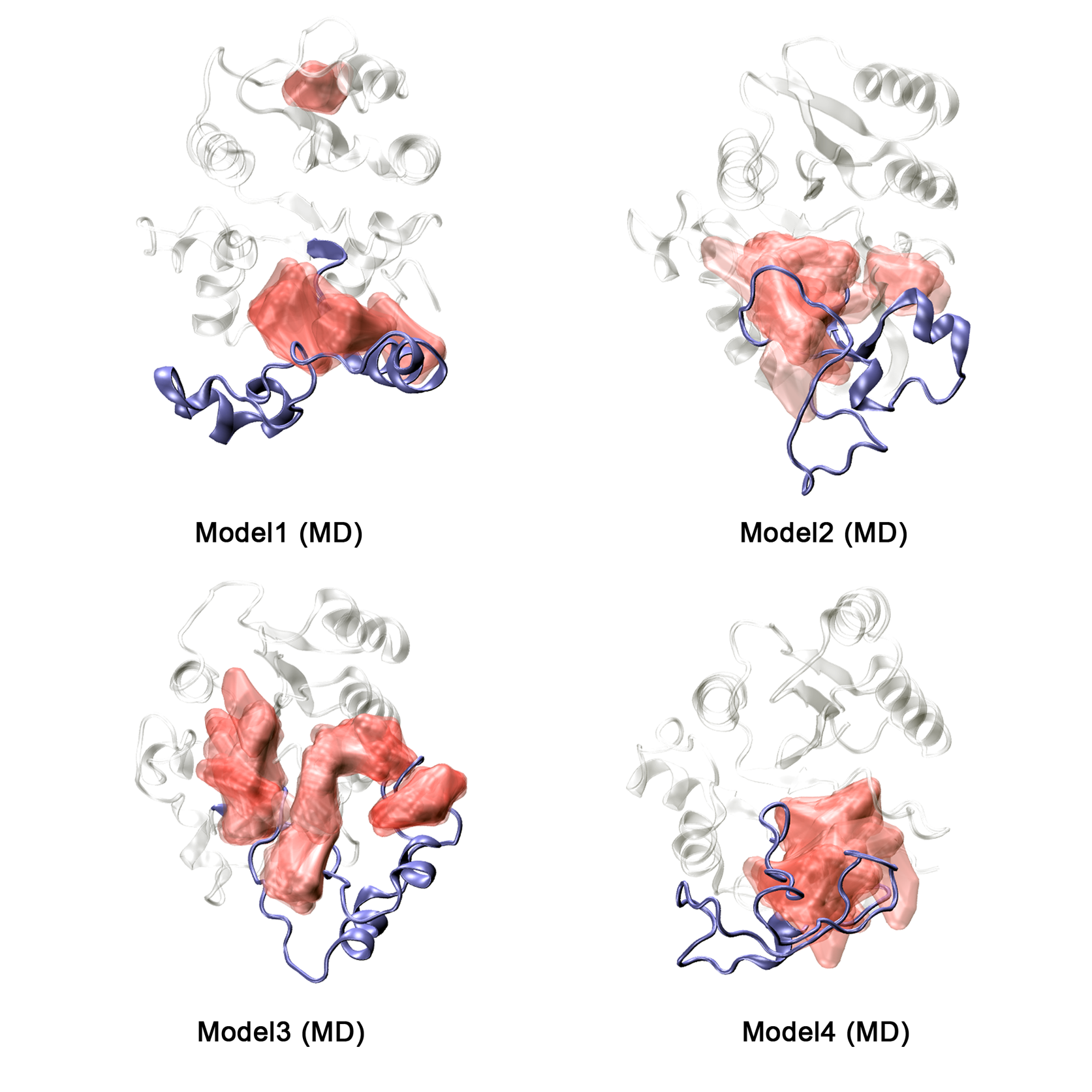

Supplement: Figure S5 — Docking of dipropionylglycerol to the four structural models after MD simulations. The most energetically favored positions are showed in red, all of them close to the variable region (marked in blue in the structures). Model 1 (-5.3/-5 kCal/mol), Model 2 (-5.8/-5.4 kCal/mol), Model 3 (-5.8/-5.4 kCal/mol), Model 4 (-5.7/-5.4 kCal/mol). (TIF) [file pone.0081990.s006.tif]
