# Supplementary material for: Structure-Function Features of a Mycoplasma Glycolipid Synthase Derived from Structural Data Integration, Molecular Simulations, and Mutational Analysis
Source: PLoS One. 2013 Dec 3;8(12):e81990. doi: 10.1371/journal.pone.0081990 (PMC3849446; doi:10.1371/journal.pone.0081990)
Supplement: Table S1 — Residues of GT MG517 (Nt GT domain, aa 1-220) located at <4Å from the UDPGlc donor in the four structural models. The number of structures out of 100 structures generated per model (see Material and Methods) are given. Highlighted residues are those selected for mutagenesis experiments. (PDF) [file pone.0081990.s007.pdf]

Table S1. Residues of GT MG517 (Nt GT domain, aa 1-220) located at <4Å from the UDPGlc donor in the four structural models. The number of structures out of 100 structures generated per model (see Material and Methods) are given. Highlighted residues are those selected for mutagenesis experiments.

Res	Model1	Model2	Model3	Model4	Res	Model1	Model2	Model3	Model4	Res	Model1	Model2	Model3	Model4
**10**	100	100	100	100	**77**	20				**169**	3			
**11**	100	80		50	**93**	100	100	100	100	**170**				10
**12**	100	100	100	100	**94**	100	100	100	100	**171**	40	70		
**13**			10		**95**	90	100	40	100	**172**		70		
**14**				10	**125**				50	**190**	6	7	14	7
**40**	10	40	100	10	**126**				60	**191**		8	22	8
**68**	3	6	3	4	**127**				20	**192**	58	68	81	63
**69**	4	5	8	2	**128**				20	**193**	69	56	99	65
**70**			53		**137**	6				**194**	16	2	100	8
**72**			100		**138**	11				**218**			10	
**73**	100	100	100	100	**139**	10				**219**	10	20		30
**76**		30	100	50	**168**	2		23		**220**	80	80		50
