# Supplementary material for: Structure-Function Features of a Mycoplasma Glycolipid Synthase Derived from Structural Data Integration, Molecular Simulations, and Mutational Analysis
Source: PLoS One. 2013 Dec 3;8(12):e81990. doi: 10.1371/journal.pone.0081990 (PMC3849446; doi:10.1371/journal.pone.0081990)
Supplement: Table S2 — Summary of MD simulations (PDF) [file pone.0081990.s008.pdf]

Table S2. Summary of MD simulations and clustering.

Hybrid structures	Stationary state (ns) [a]	RMSD cutoff (nm) [b]	RMSD ranges (nm) [b]	Average RMSD (nm) [b]	RMSD Cluster selected (nm) [b]	RMSD Frame selected (nm) [b]	Selected representative structure [c]	Helix converged [d]
Model1	850 – 1000	0.12	0.061 - 0.33	0.181	0.127	0.112	986.4 ns	S123-D133
Model2	800 – 1000	0.12	0.051 - 0.326	0.151	0.123	0.105	879.2 ns	Y126-D133
Model3	600 – 1000	0.13	0.061 - 0.700	0.265	0.136	0.116	646.9 ns	K131-K137
Model4	750 – 1000	0.14	0.061 - 0.568	0.135	0.135	0.114	960 ns	C128-K131

[a] Trajectory used for cluster analysis. Starting point selected when the backbone RMSD fluctuations were <0.5 Å. [b] Cluster analysis. [c] Structures selected as representative models by MD. [d] Position of the α-helix converged in the variable region.
