# Supplementary material for: Structure-Function Features of a Mycoplasma Glycolipid Synthase Derived from Structural Data Integration, Molecular Simulations, and Mutational Analysis
Source: PLoS One. 2013 Dec 3;8(12):e81990. doi: 10.1371/journal.pone.0081990 (PMC3849446; doi:10.1371/journal.pone.0081990)
Supplement: Table S3 — Mean RMSD and RMS fluctuations (in parenthesis) for the full structure and selected amino acid residues. Donor movement during the MD simulations. (PDF) [file pone.0081990.s009.pdf]

Table S3. Mean RMSD and RMS fluctuations (in parenthesis) for the full structure and selected amino acid residues. Donor movement during the MD simulations.

	Model 1	*Model 2*	*Model 3*	*Model 4*
**RMSD (Å)**				
Full protein	3.5	4.25	4.3	3.15
Y12	4.8 (1)	2.7 (1)	3.8(1)	2.4(1)
D40	3.5 (1)	**6.2 (1)**	3.7(1.2)	2.4(1)
Y126	4.9 (0.6)	3.2 (0.5)	**10.7(0.7)**	**10(1.7)**
F138	**7.5**	**7.5**	**17.7**	**18.2**
Y169	1.7 (0.6)	4 (0.6)	3.4(1.4)	2.3(2)
I170	2.4 (0.6)	**5.8 (0.6)**	3.1(0.8)	2.7(2.4)
W171	3.2 (0.6)	**6.1 (0.7)**	1.33(0.7)	3.4(2.2)
E193	3.1 (1.4)	**8.8 (1)**	**9.7(1.6)**	2.3(1.4)
D194	3 (1)	4.2 (1)	**9.1(1.5)**	1.6(1.2)
**Distance (Å)**				
Y126-Y169 (M)	9.6	15.9	18.9	9.5
Y126-Y169 (MD)	9.8	13.8	11.8	13.6

The residues selected for site directed mutagenesis were analyzed by RMSD in the final structures after MD simulations to test the drift they had individually relative to the initial modeled structures. The residue that deviates more is Phe138, due to the rearrangement of the variable region during the MD simulations. The rest of the residues keep an average RMSD of 4.3 Å. Larger values were seen for Glu193 and Asp194 in Model 3, due to their location in a non-structured region just previous to helix α6. Another displacement is visible for Tyr126 in models 3 and 4, probably as a consequence of the variable region rearrangement. Fluctuations along the stationary state of the trajectories (in parenthesis in Table S4) were small. The distance between Tyr126 and Tyr169, notably different between models (M in Table S4), converged to a value of 12 ± 3 Å in the final structures after MD simulations (MD in Table S4).

Donor movement during the MD simulations

UDPGlc suffers, along the MD simulations, an oscillating movement inside the pocket that results in the disruption of the interaction between N3 of the uracil ring and the residue D40. As a consequence, the UDPGlc substrate deviates from the proper position in the binding pocket seen in other crystallized GT-A/donor complexes. Docking experiments (results not shown) with rigid and flexible residues, applied to the final structures from the MD simulations did not recover the original position of UDPGlc. A closer analysis shows that the binding pocket suffers some collapse probably due to the lack of the C-terminal part of the protein in the models. The donor is rather accessible to the solvent, but it might be partially covered by other structural elements from the C-terminal part of the protein, not included in the modeled structures.
