# Supplementary material for: Structure-Function Features of a Mycoplasma Glycolipid Synthase Derived from Structural Data Integration, Molecular Simulations, and Mutational Analysis
Source: PLoS One. 2013 Dec 3;8(12):e81990. doi: 10.1371/journal.pone.0081990 (PMC3849446; doi:10.1371/journal.pone.0081990)
Supplement: Table S4 — Primers used for SDM-PCR. (PDF) [file pone.0081990.s010.pdf]

**Table S4.** Primers used for SDM-PCR.

| | |
|---|---|
| Y12A sense | 5’-GTTCCTTGTGCCAAATCAAAACC-3’ |
| Y12A antisense | 5’- GATTTGGCACAAGGAACTAATATACTAAC-3’ |
| Y12M sense | 5’-GTTCCTTGTATGAAATCAAAACC-3’ |
| Y12M antisense | 5’-GATTTCATACAAGGAACTAATATACTAAC-3’ |
| D40A sense | 5’-TTCAATGGCAATGTTGCTGATG-3’ |
| D40A antisense | 5’- AACATTGCCATTGAAAAAAATAATTTTAG-3’ |
| D40K sense | 5’-TTCAATAAAAATGTTGCTGATGAAACC-3’ |
| D40K antisense | 5’- CAACATTTTTATTGAAAAAAATAATTTTAGC-3’ |
| Y126A sense | 5’-GTATGGTCGCCTTATGCTTCTTAAAAC-3’ |
| Y126A antisense | 5’-GCATAAGGCGACCATACTTTTTAATACAC-3’ |
| Y126F sense | 5’-GTATGGTCTTTTTATGCTTCTTAAAAC-3’ |
| Y12F antisense | 5’-GCATAAAAAGACCATACTTTTTAATACAC-3’ |
| F138A sense | 5’- CATTATTAAAGCTTTGCCTTTAAAA GGT-3’ |
| F138A antisense | 5’- AAGGCAAAGCTTTAATAATGAAATCAT-3’, |
| Y169A sense | 5’-GATCAAGCCATTTGGAATATTGTTATAAAC-3’ |
| Y169A antisense | 5’-CCAAATGGCTTGATCATTATTTTTG-3’ |
| I170A sense | 5’-GATCAATATGCCTGGAATATTGTTATAAAC-3’ |
| I170A antisense | 5’-TATTCCAGGCATATTGATCATTATTTTTG-3’ |
| W171A sense | 5’-GATCAATATATTGCCAATATTGTTATAAACACAG-3’ |
| W171A antisense | 5’-CAATATTGGCAATATATTGATCATTATTTTTG-3’ |
| W171G sense | 5’-GATCAATATATTGGCAATATTGTTATAAACACAG-3’ |
| W171G antisense | 5’-CAATATTGCCAATATATTGATCATTATTTTTG-3’ |
| E193A sense | 5’-GTTATTTGCCGATATACCAATCTGG-3’ |
| E193A antisense | 5’-GTATATCGGCAAATAACCTTGATTC-3’ |
| D194A sense | 5’-TTTGAAGCCATACCAATCTGGTATCC-3’ |
| D194A antisense | 5’-TTGGTATGGCTTCAAATAACCTTGATTC-3’ |
| Y218A sense | 5’-GAACAAATGCCTTTATTCGTAATGATAGTTTATC-3’ |
| Y218A antisense | 5’-CGAATAAAGGCATTTGTTCCTATCACATC-3’ |
